# Finding an existential place to rest: enabling well-being in young adults

**DOI:** 10.1080/17482631.2022.2109812

**Published:** 2022-08-07

**Authors:** Maria Lundvall, Lina Palmér, Ulrica Hörberg, Gunilla Carlsson, Elisabeth Lindberg

**Affiliations:** a Faculty of Caring Science, Work Life and Social Welfare, University of Borås, Borås, Sweden; b Department of Health and Caring Sciences, Linnaeus University, Växjö, Sweden

**Keywords:** Existential concerns, reflective lifeworld research, resilience, phenomenology, qualitative research, young adults

## Abstract

What enables well-being when experiencing existential concerns as a young adult is an under-explored area of research. In order to address young adults’ existential concerns and provide caring support that builds their resilience to meet life challenges, the purpose of the study is to describe the meaning of enabling well-being as experienced by young adults living with existential concerns. This phenomenological study is based on a reflective lifeworld research. Seventeen young adults, aged 17–27 years, were interviewed. The results is presented in an essential meaning and further explored with its variations and individual nuances of the phenomenon; enabling well-being. The essential meaning of enabling well-being, when experiencing existential concerns as a young adult, means finding a place to rest. Finding a place to rest means finding both movement and stillness in life to reflect upon one’s life story in order to understand oneself. The results also show that young adults enable their own well-being in many ways when experiencing existential concerns. When their existential concerns feel overwhelming, they need support from healthcare professionals. When young adults seek professional support, the professionals must be open and focus on the young adults’ life story to enable well-being.

## Introduction

Caring science deals with existential aspects of human life, and addresses issues such as human vulnerability and search for meaning. As such caring science can act as the starting point when describing what enabling well-being in young adults. This study focuses on the meaning of enabling well-being in young adults^1^ daily life in Sweden who are experiencing existential concerns; it is part of a larger research project studying young adults living with existential concerns and who have sought support

According to Arnett (2001), the time as a young adult, between 16–29 years of age, is a “emerging” to become an adult. It is a special and transformative period in life. It is a time of many changes and a time of exploring the outside world to become an independent adult. To grow from child to an adult is a period of identity exploration, exploration of sexuality, instability, self-focus and attempts to create meaningfulness while at the same time becoming independent and developing the ability to handle various events in life (Kinnunen et al., 2010). It is a time when young adults also develops socially and emotionally. Their thinking develops and there are physiological changes (Frydenberg, 2008). Attitudes towards school, work, parents and friends change (Hwang et al., 2018). The opportunities to handle change can depend on various factors such as access to work, friends, housing and financial conditions (Frydenberg, 2008). Relationships with other people, such as family and friends, are also of great importance (Wannebo et al., 2018). For most young adults, this change is positive and they feel that they grow as human beings but for some young adults the transition involves a sense of inability to cope with their existence (Byrne et al., 2007; Hwang et al., 2018).

The transition from childhood to becoming a young adult can be experienced as touching an existential level of being. In some situations, this includes feelings of worries, anxiety and a search for meaning and may lead to existential concerns in a young adult (Lisznyai et al., 2014). Worry, anxiety, and stress are normal experiences and are important attributes when meeting life’s challenges (Yalom, 1980). However, feelings of excessive worry, anxiety, or stress can have negative consequences if they are not noticed or addressed. In young adults, such excess can cause them to fall behind in their schoolwork, experience degraded social functioning, and suffer poorer quality of life (The National Board of Health and Welfare, 2017). The loss of well-being and deteriorating quality of life without biological reason can be seen as a life crisis. Not having control over life is often a horrible and unexpected experience. The key to getting well again is to get resources and help, which most people experience with shame and guilt. It is very important for caregivers to support well-being to prevent existential disaster, suicide or severe depression (Ventegodt et al., 2005).

Existential concerns such as thoughts about life, who we are, and what we wish to be are all part of human life. Existential concerns can be triggered, for example, when a person becomes ill or is confronted with death or another person’s illness. These concerns can also develop during the transition from childhood to young adulthood as well as throughout other life phases. When everyday life is challenged, thus we begin to think about our existence and what it means to be a human being (Yalom, 1980), causing us to reflect on the meaning of life on a deeper level and have doubts about our existence. In terms of young adults, these existential concerns can grow unmanageable and disrupt their daily lives, manifesting as anxiety, stress, and depression (Besharat et al., 2020; Moore & Goldner-Vukov, 2009). Young adults experiencing existential concerns try to understand how life should be handled and how life can be meaningful. They can feel an intense longing to share their existential concerns with others whilst being afraid of being vulnerable through such sharing. While this longing indicates a willingness to feel harmony with oneself, it can feel unachievable, creating feelings of hopelessness and loneliness (Lundvall et al., 2020, 2019).

To support and strengthen the health and well-being of young adults with existential concerns, it is proposed that healthcare professionals create spaces in which young adults can dwell without feeling vulnerable (Lundvall et al., 2020, 2019). In a mental health context, support has been described as using health-promoting strategies to strengthen individuals’ autonomy and participation in care, to improve their quality of life (Seow et al., 2016). Regarding young women’s health and well-being, Larsson et al. (2012) emphasize the importance of creating forums in which young women can talk about their situations. Research shows that adolescents with self-harming behaviours often turn to friends with their problems, as they find it difficult to confide in and seek support from healthcare professionals—they need adults but lack them as resources for support (SBU, 2015). There are several challenges and obstacles for young adults to seek support for their ill-being. There are fears of seeking support and of not being taken seriously (Goodwin et al., 2016; Yap et al., 2013). There is also a fear of seeking support for something that is considered normal and therefore the young adults try to solve their problems themselves (Bluhm et al., 2014). Other reasons why young adults do not seek support are that there is a tendency to stigmatize mental disorders (eg depression, anxiety and worry) in society (Gulliver et al., 2010). Another problem is that young adults do not know where to turn with their suffering (Åsbring & Hochwälder, 2009). All young adults, but especially vulnerable ones, need to talk about their lives, and they need to be listened to as individuals if they are to feel hope about their futures (Lindgren et al., 2011).

The World Health Organization (WHO, 2012) has stressed the importance of youth-friendly receptions and has developed a national quality standard for all countries to use to make it easier for young adults to obtain the health services they need to support and improve their health and well-being. However, a pronounced focus on recognizing and supporting existential concerns is less prominent. For example, Sweden supports children’s and young adults’ health needs via departments in each municipality that offer preventative care, supportive care, or treatment care (Wieselgren, 2018). However, research shows that the design of youth-friendly services is not comprehensive, and more has to be done (Tylee et al., 2007).

From a caring science perspective, the role of healthcare professionals is to support and strengthen patients’ health processes towards improved health and well-being (Dahlberg, 2018). Dahlberg and Segesten (2010) and Galvin and Todres (2013) argue that health is achieved when patients’ health processes are strengthened and patients regain balance in their lives such that they can fulfil both small and large life projects. Galvin and Todres (2013) believe that existential suffering can manifest in many different ways depending on patients’ experiences and their beliefs about creating meaningfulness in existence. Empirical research on how to strengthen health and well-being in young adults reveals that talking about their lives and experiences may be greatly beneficial (Larsson et al., 2012). From a lifeworld perspective, care should be focused on individuals’ uniqueness beyond just medical symptoms, enabling them to receive appropriate support to strengthen their health processes (Todres et al., 2014). Health from a lifeworld perspective involves the whole person and involves a sense of balance and equilibrium in relation to life and the people in close proximity (Dahlberg, 2011; Galvin & Todres, 2011).

What enables well-being when experiencing existential concerns as a young adult is an under-explored area of research. In order to address young adults’ existential concerns and provide caring support that builds their resilience to meet life challenges, the aim of the present study is to describe the meaning of enabling well-being as experienced by young adults living with existential concerns.

## Design and method

This phenomenological study is based on a reflective lifeworld research (RLR) approach described by Dahlberg et al. (2008). RLR finds its epistemological basis in phenomenological and hermeneutical philosophy as described by Husserl, 1936/1970; Husserl, 1929/1977), Merleau-Ponty (1962/2002) and Gadamer (2013). RLR is phenomenon-oriented and characterized by the phenomenon being in focus during the whole research process. In the present study, the phenomenon is *enabling well-being* as experienced by young adults living with existential concerns who have sought support in health care as well as from family members or others in their immediate environment (e.g., friends) and their school environment. Founded in the described philosophy, the methodological principles of RLR are openness, flexibility, and bridling one’s own understanding of the phenomenon being studied. Bridling one´s own understanding means asking reflective and critical questions about the material during the process to avoid taking for granted what is unknown (Dahlberg et al., 2008). These methodological principles are used throughout the research process to grasp and describe the phenomenon.

## Participants

Seventeen young adults, nine women and eight men aged 17–27 years, were included in the study (Table 1). The participants were all native Swedes from the south of Sweden. They lived in either rural areas or in a big city. The young adults worked, studied, or were on sick leave. Inclusion criteria were that participants were aged 16–25 and had a lived experience of existential concerns for which they had sought support. The places or people from whom they had sought support were part of the healthcare system, school healthcare facilities, family members, or close friends.

**Table 1.** Participants.

Interview no	Gender	Age
1	Female	25
2	Male	23
3	Female	17
4	Female	24
5	Male	22
6	Female	23
7	Female	18
8	Female	19
9	Female	25
10	Male	24
11	Female	18
12	Female	21
13	Male	24
14	Male	27
15	Male	24
16	Male	21
17	Male	24

Recruitment was twofold. First, healthcare professionals from different healthcare centres asked young adults if they were interested in knowing more about the study. If they did, these professionals gave the participants’ telephone numbers to one of the researchers (ML). The researcher (ML) then contacted each participant to provide her or him with more information about the study. The second recruitment method involved advertising the study via posters placed at various healthcare centres as well as digitally posting them on media channels. The young adults contacted the researcher (ML) directly for more information about the study. One participant who showed interest in participating was over 25 years old but had experienced existential concern since adolescence. The researchers decided to include the young adult, as the lived experiences were a crucial part of the phenomenon under study and deemed it ethically correct to allow the person to share experiences of existential concerns as a young adult instead of excluding this person due to age. In connection with the first contact, if needed, the phenomenon existential concern was clarified.

## Data collection

Seventeen audiotaped lifeworld interviews were conducted by the first author according to the principles of the RLR approach (Dahlberg et al., 2008). Each interview started with an open question to invite the participant to speak freely about the phenomenon; “You have sought support for your existential concern. Could you tell me about it?” During the interview, the interviewee asked follow-up questions to further explore the phenomenon, such as “Can you give an example of … ? How did that make you feel?” One interview took place in the participant’s home environment, whereas the remaining interviews took place at the university according to the participants’ wishes.

Lifeworld interviews are a suitable way to explore lived experiences and to grasp a phenomenon (Dahlberg et al., 2008). During the interviews in the present study, the researcher tried to remain open and compliant with how the phenomenon presented itself by asking follow-up questions to elucidate any variations in the phenomenon’s meaning. At the same time, the interviewer intentionally lingered on the participants’ answer and did not move too quickly in terms of asking new questions.

## Data analysis

The data were explored and analysed to determine the meaning structures that describe the phenomenon of enabling well-being. The method principles described by Dahlberg et al. (2008) were followed throughout the analysis process. The aim of analysis is to describe the essence of the phenomenon under study and its constituents. An essence is defined here as a structure of meanings that describes the phenomenon and the essence is what makes the phenomenon the phenomenon (Dahlberg, 2006). The entire analysis process can be understood as a movement between the whole (interviews) and the parts (meaning) as well as a new whole (essential meaning).

The data analysis began by first reading the interviews several times to open the researchers’ minds to the possible meanings contained in the data. This was done to acquire an overall understanding, which was required to move freely between wholes (interviews) and parts (meanings). The analysis continued by searching for meanings—meanings that seemed to belong together were grouped into clusters. The analysis was continued by looking for how the clusters were interconnected in a search for patterns of meanings. Movement between the parts and the whole was done to gain a deeper understanding of the phenomenon and to describe its essential meaning structure. The essential structure speaks of how the meanings relate to each other and is the most abstract level (Dahlberg et al., 2008; Van Wijngaarden et al., 2017). After this description, the analysis process continued to identify variations and nuances within the phenomenon; *enabling well-being*. Constituents are understood as parts of the pattern and meaning structure of the essence. The constituents give examples of how the phenomenon manifests itself at the meaning level, which is concretized with the support of quotations (Dahlberg et al., 2008; Van Wijngaarden et al., 2017). Throughout an analysis process, it is important to maintain openness and a bridled attitude to allow the phenomenon to be uncovered; this also means not understanding meanings too quickly. By asking reflective and critical questions to the data, such as “Why do I understand it as … ? Could it be understood as something else?” In so doing, the researchers preserved open and bridled attitudes and maintained flexibility towards the meanings that emerged throughout the analysis. The results section first presents the essential meaning and then describes the meanings that further constitute the phenomenon with its variations and individual nuances of the phenomenon; *enabling well-being.*

## Ethical considerations

The study followed the research principles described in the Helsinki Declaration (World Medical Association Declaration of Helsinki, 2013) and the Swedish Research Ethics Guidelines (Ministry of Education, 2003). Ethical approval was also obtained from the Ethics Review Board in Gothenburg (Dnr: 483–16). An approved supplementary application for how to reach participants was also secured (Dnr: T322-18). One ethical aspect of this study that was carefully considered was that young adults with existential concerns are a vulnerable group of people both in that they are young and that they are experiencing existential concerns. Another ethical consideration was that during the interviews, the young adults might reveal things that they had not previously told anyone. Therefore, it was crucial that after the interviews, if informants expressed the need to talk about their situations further, the interviewer, a public health nurse, stayed on to continue the discussion and/or offered to set up a meeting with another healthcare professional with the ability to handle existential conversations. It was important to ensure the participants were fully aware of the voluntary nature of their participation, and that they had the right to cease participation at any time. All informants were informed, both verbally and in writing, of the voluntary nature of the study and his or her right to cease participation at any time without giving an explanation.

## Results

The results section first presents the essential meaning (what makes the phenomenon this very phenomenon) and then describes the meanings that further constitute the phenomenon with its variations and individual nuances of the phenomenon; *enabling well-being.*

The essential meaning of enabling well-being, when experiencing existential concerns as a young adult, means finding a place to rest. `Finding a place to rest’ means to find a place in which recovering from life challenges can be made. Such a place offers space to put the puzzle of life together and come to terms with life’s challenges. Finding a place to rest means finding both movement and stillness in life to reflect upon one’s life story in order to understand oneself. When understanding of life increases, it enables movement in which the rhythm of life is supported and recovery is enabled. A rhythm in life means finding harmony in life that enables personal growth and finding peace with existential concerns. Reflecting one’s life story means gaining an increased understanding of life and the emotions that arise when experiencing existential concerns.

Reflection is a process that occurs both alone and with others as a shared experience. It takes time to realize that there is a need to share one’s existential concerns with others to deal with life’s challenges, and young adults experiencing existential concerns tend to reflect in solitude to handle substantial life situations. Well-being can emerge when reflecting in solitude, but existential concerns can also become unbearable, and the need to share one’s life story with someone else can emerge. Relationships permeated with openness towards the life story is crucial. By inviting, and being invited, into a relationship where there is an opportunity to share the life story, hope is gained that life can continue and that there is a meaningful future ahead. When existential concerns is shared with a trustworthy person, it supports an increased understanding of how life’s sometimes difficult challenges can be balanced and enabled one´s own ways of living. If the life story is not heard or recognized, young adults can develop feelings of rejection, disrupting life’s rhythms.

The essence is further described in the following constituents: recovering in solitude, sharing one’s life story in everyday life, and reflecting one´s life story in a trusting and caring relationship.

## Recovering in solitude

Recovering in solitude means that young adult, when experiencing existential concerns, tries to understand their own existence to enable well-being. Recovering in solitude means trying to understand and deal with life’s challenges in order to feel hope that life can improve. Reflecting in solitude is a challenging and laborious process towards understanding that require young adults to dwell on their existential concerns and re-evaluate who they have become in order to understand their life situations: “I like to be alone, when I choose to be alone. I think it is very important to get to know oneself and to think for oneself (7).”

Recovering in solitude also means harbouring the existential concern that constantly demands attention; this includes both movement and stillness to enable well-being. In stillness, reflecting in solitude can mean being creative. For example, writing stories, poetry, or a diary. Such creative acts helps to work through emotions and creates positive emotions from the fact that they succeed in a task, which enables well-being.

It is a freedom—you simply forget yourself for a moment. And you can live in a world you have created … It gives me the chance to gather thoughts, really bring up ideas that I have, philosophical thoughts you know, such things [like] (16).

Recovering in solitude includes letting thoughts and feelings rest by withdrawing from others. In solitude, thoughts are allowed to rest by focusing on something completely different such as watching movies, listening to music, or playing computer games. Here, the young adults takes a break from the outside world and has a chance to recover.

[I] turn on any movie or turn on any sound only. I do not need to watch it, but I want it to be there and then I’m calm for an hour and a half because I do not have to think about anything else during this hour and a half (8).

Here, recovering in solitude means there is movement to find strategies that enables well-being in daily lives. A place to rest also means taking refuge from concerns and living in the present to enable well-being. For example, having responsibility for an animal means being present and only concentrating on taking care of the animal.

It has been the horses that have helped me. I have to be in the present in some way, and I cannot really think about everything, both forwards and backwards—I have to be here and now (9).

In this state, the young adults reflect upon difficult situations they had faced during the day and tries to determine different ways to handle similar situations in the future. This solitude also enables well-being by simply allowing them to be themselves; however, this solitude also increases the risk that the existential concerns becomes magnified.

When young adults no longer cope with existential concerns on their own, they need to talk about their situation with others to gain inner balance and peace of mind. To gain peace of mind in the most radical way, some consider ending life. In this overwhelming situation, and at the absolute bottom, there is access to an openness to share the life story with others:

Then, I felt that I had no choice anymore. It was like I had to talk to someone or take my life … those were the choices I had (10).

## Sharing one’s life story in everyday life

Sharing one’s life story in everyday life, as a young adult experiencing existential concerns, means opening up and sharing the innermost selves with someone close to enable well-being. This sharing is a movement in life that helps coping with existential concerns. In the act of sharing, young adults select pieces of their life story to family and friends enabling them go about their daily life. This means that the burden of the existential concerns eases. When pieces of the existential concerns shares, a stillness occurs. Here, stillness means not having to fight in solitude anymore and enables everyday life to flow with more ease.

When I get caught up in my own “brain ghosts,” someone needs to just calm me down. Just tell me, it will be fine. I cannot tell myself that, it is not possible … [It helps] to know that I have someone’s support (3).

Young adults with existential concerns is provident with whom and how much they share of their existential concerns; this caution reduces the risk of being condemned, rejected, or disliked. A trusting relationship has to establish before they share their existential concerns. It takes courage to show vulnerability, and a trusting relationship enables to expose their innermost thoughts: “I did not know who I could trust, I felt incredibly alone. If I did not have my sister, I probably would not have been sitting here. I trust her one hundred percent (2).”

Sharing existential concerns in a trusting relationship is a balancing act of how much of the existential concern to share to avoid over-burdening close friends and family members. Young adults with existential concerns often face difficulties communicating the deepest and most difficult thoughts, as they risk hurting or disappointing their loved ones. Out of care for the person receiving the existential concern, they carefully chose what and how much to share:

I don’t want to be the one who is sad all the time or always put a burden on mom. And even though we talked a lot about it, and she knew I was sad, it felt like it was easier to talk to someone else (11).

Young adults feel a closeness and security with close friends, which enables them to share their existential concerns. This closeness and security lies in the fact that the friends also experience similar problems and understand the situation; however, it also feels as though their friends’ knowledge of life’s sometimes-difficult challenges are as limited as their own. Thus, sharing one’s life story with close friends may not always enable the necessary movement towards well-being. When their life rhythm is disrupted, the need for further reflection in a caring relationship emerged: “Mom also knows a lot, and I still talk a lot with her today. It is not that I stopped sharing with my mother and went to a psychologist, but I just kind of split it up a bit (7).”

## Reflecting one´s life story in a trustful caring relationship

In contrast to reflecting in solitude, reflecting in a trustful caring relationship, means being able to put into words what feels difficult and challenging together with an educated and trusted person. Reflecting with those who are not familiar with the situation or know the young adult can feel like a relief: “Here was more like I could talk completely freely, I could express myself for the first time without anyone saying that it was right or wrong (12).” At the same time, it can be challenging to share existential concerns with someone who do not know the young adult. There is always a risk of being neglected or trivialized, of not being heard, or in the worst case, of not being taken seriously: “It is hard enough to realize that you feel bad and that you need help, and then when you stretch out a hand, you get hit in the face—then it becomes even more difficult (13).”

When young adults with existential concerns finally is able to contact professional care to enable well-being, they feel gratitude at meeting someone who can help their understanding process. However, not getting, or having to wait, for support when they have summoned the courage to try to understand their life situation with someone else means increased hopelessness, well-being seems further and further away.

I called the healthcare center and I got “ah, you have to wait two to three months.” Once you are at the bottom, there is no alternative. It was—it was not an emergency, but it was still urgent (5).

When experiencing existential concerns, young adult’s fear to see themselves through someone else’s perspective, as it could show sides of themselves that were not previously visible. In joint trusting reflections, a more forgiving description of themselves and their life situations can emerge, as new and open questions arise: “It was as if she was listening and then she asked questions and follow-up questions that allowed me to go into myself—to see myself from a new perspective and see things that I had not seen before (14).” This novelty allows young adults to talk about their existential concerns in new ways, using new words and descriptions to illuminate existential concerns.

I thought that I understood this lady very well … we connected at once, and it felt like she, even though she was in a different generation than I am, she understood me. She tried to listen to me and not offer up a lot of exercises and stuff but just … talked and tried to understand my problems in life (17).

Reflecting one’s innermost thoughts in trusting relationships creates the possibility of movement in life. With this movement, young adults can be true to themselves and find their own solutions. Finding one’s own strategies feels genuine, and feels it is possible to find the genuine self, which enables well-being. In contrast, when young adults are treated as “cases” (objectified) and receives advice or strategies based on standardized templates, they feel objectified and guided by the system/care guidelines instead of being an individual. Demands for performance arises and making it more difficult to enabling well-being. It also increases feelings of hopelessness and belief that nothing can enable well-being: “ … it was as if they already decided what my treatment was before they had even asked me what … what I thought was wrong … And I became so uncomfortable with it, I do not know, it was like she would lead me somewhere [I didn’t want to go] (6).”

For young adults experiencing existential concerns the willingness to reflect one’s life story in trusting and caring relationships depends on previous experiences. For example, if rejected or condemned in previous situations, means prolonged time to open up about one’s innermost thoughts with another person and making well-being seem inaccessible. If well-being perceives as inaccessible, life seems trapped in a maze, which young adults has to navigate through:

… she had to have an exact problem [to encompass] what I felt, and I did not know what the problem was and she did not ask good counter-questions either to what I searched for … then I just stopped going to her … . It took a while before I went to someone else (7).

Being meet with templates and guidelines for grading the degree of ill-being complicates the understanding of life situation and reflection becomes absent—it merely confirms what young adults experiencing existential concerns already feel. When the grading lead to support being given elsewhere (than where they had the appointment) to get “the right” support, increased feelings of uselessness occurs. There is also a risk young adults blames themselves for being too ill, feeling that enabling well-being is more distant than before. “I do not think I was prepared to hear it … couldn’t she have let me see her a few times and let me realize that is actually serious … before she referred me further? Build more trust? (1)”

Reflecting on one´s life story within trusting caring relationships creates a sense of security, knowing that you have someone to return to and to continue reflecting with about existential concerns, supports life rhythms and enables well-being.

## Discussion

This study focuses on the phenomenon of enabling well-being as it is experience by young adults with existential concerns. The essential meaning shows that young adults experiencing existential concerns need a place to rest in order to recover. When they find a place to rest they have space to put the puzzle of life together and feel strong enough to come to terms with life’s challenges. A place to rest is more than just a physical room—it is a place where their recovery is supported and where they are given the space to tell and dwell upon their life stories.

The results shows that young adults strive for well-being in many different ways; in solitude, with family and close friends. Once they seek support from healthcare professionals, there is a driving force in trying to understand themselves and the importance of a place to rest to enable well-being. To understand the significance of place to rest when experiencing existential concern, we can take support from the philosopher Heidegger’s ontological ideas about the existence of humans being in the world. Heidegger (1927/2013) describes humans exists in a context and interacts with others, which affects humans in himself and in relation to others. In this context, both opportunities and limitations of being in the world are raised. According to Heidegger (1927/2013), freedom does not mean that there is absolute freedom, but rather that freedom is limited. It means limitations in relation to human vulnerability, such as: the fact of the finitude and death of life, the fragility of our bodies, that man lives in a certain time, in a specific place with its culture and language. Heidegger (1927/2013) describes that we as humans have a responsibility to manage our lives in the best way and not just follow the masses and thoughtlessly let ourselves go here and there. Kåver (2017) illuminates that it can be scary for humans to stop and reflect on the meaning of life, see all the possible choices and obstacles that may exist. From this reasoning, we can understand that young adults are free to find their own way, but opportunities and choices also mean difficulties in choosing direction of life. The young adults must realize that they are becoming through the way of relating to life. Freedom, choice and responsibility are examples of what all people are faced with and an insight into the existential dimensions of life helps human to make authentic life choices (Van Deurzen, 2009).

Philosophy also helps us to understand the meaning of place as something more than a physical room. Husserl (1940/1981) holds that the body is the core from which humans perceive their place and from which everything else is given a place and a direction. This means that we identify and orient ourselves upon a site as a starting point. Merleau-Ponty (1962/2002) further develops Husserl’s ideas about the body as lived. The body is the opening to the world with which human beings communicate with others and explore their surroundings. We are dependent upon our bodies to explore the world, interact and grow with other people, and understand ourselves and others. Galvin and Todres (2013) describe “being human” as coming from a certain place, a place that is not only a physical living environment but also where the feeling of being at home becomes meaningful. To be at home means to feel a sense of belonging, familiarity, security, and unreflective ease. From this reasoning, we can understand that finding a place to rest means that young adults existential concerns, thoughts, and feelings can get the space needed to be seen and reflected upon in solitude or with friends, close family, and healthcare professionals. Based on this reasoning, it is important to encourage young adults with existential concerns to open up about their lived experiences—to talk about and reflect upon their life stories in order to strengthen their well-being and strengthen their ability to make authentic choices.

From a caring science point of view, an important starting point is to try to understand the lifeworld of young adults in order to strengthen their health processes and give them the courage and vitality to perform their small and large life projects (Dahlberg, 2018). In solitude, young adults try to enable their own well-being by attempting to understand their situations and find strategies to deal with life. At the same time, this can cause their existential concerns to become unmanageable. By sharing their life stories, they might come to peace with their situations while taking a risk in allowing themselves to be vulnerable (Lundvall et al., 2020, 2019). Östman et al. (2020) explain that young adults strive to understand their ethos (innermost values, or the voice of their heart) and their access to health by self-managing their freedom, independence, and responsibility. This ethos can challenge healthcare professionals in encounters with young adults—caregivers must be receptive to the young adults’ life story in order to provide support in this demanding phase of becoming an adult.

Galvin and Todres (2013) description of well-being as “existential dwelling” is relevant to understanding a “place to rest.” Existential dwelling means being open and letting “what is there” (existential concerns) be present before attempting to make changes. To dwell means to listen to what is there, to stay, and to accept what is revealed. If young adults’ dwelling is supported, they can achieve a feeling of inner peace. The most important way to enable well-being through dwelling is to be there for them no matter what happens. Dwelling is intentional in its attunement—it allows the world, the body, and others to flow by being in the present, gaining support for the past, and remaining open to what the future has to offer. Dwelling allows youths to “feel at home” in themselves and in the world. From this, we understand the importance of healthcare professionals offering young adults a place to dwell to enable their well-being. However, young adults must want to dwell if they are to achieve an inner sense of peace. This is in line with Sommer and Saevi’s (2017) understanding of how support and lived space can strengthen young adults with mental health problems. They describe that support anchored in autonomy and respect for young adult’s own abilities opens up the space for their present and the future. In this space, young adults can be nourished and access the freedom to be and to become.

In order to develop the type of caring practices that enable young adults with existential concerns well-being, we need to understand that young adults and healthcare professionals are bodily beings. Both carry understanding and experiences from their lifeworld. In the relation between them, there will always be both visible and invisible aspects. Todres et al. (2014) describes the importance of an understanding of what the lived body is expressing. The body can reveal what is invisible, such as worried looks. Merleau-Ponty (1962/2002) describes human relationships as intersubjective. Through our bodies, we inhabit space and time and are part of an interpersonal whole. In the context of this study, in order to support and strengthen young adults’ well-being, healthcare professionals need to create places where the young adults can make visible what at first glance seems invisible through reflection and together with healthcare professionals. Svenaeus (2010) describes how a “rift” in our stories caused by illness or un-health requiring opportunities to tell the story in another way than before. In this way, the young adults are given the space to grow towards a new and deeper understanding of their existential concerns, which provides them with the space to recover and feel able to confront life’s challenges.

Healthcare professionals may face barriers to creating conversations with young adults about their existential concerns. For example, their own fears of what may come up in such conversations and not being sure of how to handle them could arise. This could cause healthcare professionals to simply avoid broaching existential issues with youths (Lundvall et al., 2018). Keall et al. (2014) reports that within palliative care, existential issues can be neglected due to a lack of time, a fear that the conversation will aggravate the patient’s situation, or a lack of competence regarding how to approach existential concerns. Bullington et al. (2019) emphasizes that the core of caring is the ability to be present, sensitive, and open in conversations with patients. A conversation with a phenomenological foundation can create opportunities for patients to reflect on their life situations.

If healthcare professionals simply follow templates or give standard answers, young adults will not feel supported. Professionals need to listen to young adult’s life stories with an open mind. Ekebergh and Lindberg (2020) describe how putting experiences into words can formulate a narrative and start a reflection. Thus, professionals must strive to avoid presumptive and unreflective attitudes. They can achieve this openness by being curious, responsive, and wanting to hear the young adults’ life story, enabling deeper reflection about their situations. Gadamer (2013) believes that people are reflective by nature, and that reflection takes place in relation to consciousness, understanding, and experience. Gadamer also points to an open mind as the best way to gain deeper understanding of a phenomenon. In the context of reflection, this means lingering, slowing down one’s thinking, and opening up to “otherness”. Ekebergh and Lindberg (2020) believes that in reflection, old truths are challenged with new truths that are experienced, causing new understanding and knowledge emerge. This process of understanding is central to enabling well-being that supports and strengthens health processes in young adults.

## Methodological reflections

High-quality scientific research needs to ensure objectivity, validity, and generalizability (Dahlberg et al., 2008; Van Wijngaarden et al., 2017). It can be considered a strength in qualitative research when there is both an ontological and an epistemologically well-established foundation (Van Wijngaarden et al., 2017). The present phenomenological study followed the RLR approach (Dahlberg et al., 2008) in terms of openness, bridling, and a reflective attitude towards the phenomenon (enabling well-being). In this study, openness was achieved through a bridled and reflective attitude by constantly asking reflective and critical questions about the material during the analysis to avoid taking for granted what is unknown. To ensure objectivity, the members of the research group continuously discussed their findings and reflected upon them. They also discussed the results in detail in a seminar with other researchers, contributing to the results’ strength and validity.

Van Wijngaarden et al. (2017) argue that validity is associated with meaning and generalizability, which comes from presenting the results as both a structure of meaning and in direct quotations from the interviews. In this study, the variety among the group of young adults who participated produced rich descriptions of the phenomenon, allowing the researchers to describe the phenomenon in terms of its essence and constituents to deepen the overall understanding of the phenomenon. Regarding generalizability, it must be noted that results are always contextual. The present results are transferable to similar contexts, as the results are presented as an abstract essence and are based on varied data.

## Conclusion

This study contributes important knowledge from a caring science perspective to inform caring approaches when healthcare professionals encounter young adults. If young adults can find a place to rest, they have the opportunity to create movement in their lives. The results show that young adults enable their own well-being in many ways when experiencing existential concerns. When their existential concerns feel overwhelming, they need support from healthcare professionals. When young adults seek professional support, the professionals must be open and focus on the young adults’ life story—otherwise, they risk focusing on symptoms of for example, depression and anxiety. It is in the life story that professionals can support a forward movement. From the results, it is concluded that a place to rest is vital if youths are to invite and be invited to share the life story. Here, vulnerability becomes clear—the youths’ vulnerability and also the healthcare professionals’ vulnerability. That both can live and work with their vulnerability as a “tool” seems to be a prerequisite for a genuinely caring relationship that enables well-being.

## Relevance to clinical practice

The results reveal the importance of understanding that when young adults seek support, they have already worked with and tried to understand their existential concerns for a long time. They carry with them knowledge that healthcare professionals must make use of. Further, young adults have developed coping skills that professionals can utilize to strengthen and support young adults’ health processes to enable well-being. Enabling a place to rest where young adults can be vulnerable to understand their existential concerns, as part of being human, enables young adults to find themselves and a place of belonging. Finding oneself increases resilience to life’s sometimes difficult and challenging situations.

## Note

1. In this article, we use the term “young adults” to describe young people from the ages of 16–29
